# The mechanism of low-concentration sodium nitroprusside-mediated protection of chondrocyte death

**DOI:** 10.1186/ar1705

**Published:** 2005-03-01

**Authors:** Hyun A Kim, Ki Byoung Lee, Sang-cheol Bae

**Affiliations:** 1 Department of Internal Medicine, Hallym University Sacred Heart Hospital, Kyunggi-do, Korea; 2 Department of Orthopedic Surgery, Hallym University Sacred Heart Hospital, Kyunggi-do, Korea; 3 Department of Internal Medicine, Hanyang University College of Medicine, Seoul, Korea

## Abstract

Sodium nitroprusside (SNP), a widely used nitric oxide donor, has recently been shown to mediate chondrocyte apoptosis by generating reactive oxygen species, whereas more potent nitric oxide donors do not induce chondrocyte apoptosis. The present study was performed to investigate the protective effect of a low concentration of SNP upon the cytotoxicity of chondrocytes to higher concentrations of SNP, and to elucidate the underlying mechanism. Human osteoarthritis chondrocytes were cultured as monolayers, and first-passage cells were used for the experiments. Chondrocyte death induced by 1 mM SNP was completely inhibited by pretreating with 0.1 mM SNP. This protective effect of SNP was replicated by the guanosine-3',5'κ-cyclic monophosphate analog, DBcGMP. Protection from chondrocyte death conferred by 0.1 mM SNP was mediated by heme oxygenase 1 (HO-1), as was revealed by the increased expression of HO-1 in 0.1 mM SNP pretreated chondrocytes and by the reversal of this protective effect by the HO-1 inhibitor, zinc protoporphyrin. SNP-mediated chondrocyte protection correlated with the downregulation of both extracellular signal-regulated protein kinase 1/2 and p38 kinase activation. SNP at 0.1 mM induced significant NF-κB activation as revealed by electrophoretic mobility shift assays, and the inhibition of NF-κB by MG132 or Bay 11-7082 nullified 0.1 mM SNP-mediated chondrocyte protection. The upregulation of p53 and the downregulation of Bcl-_XL _and Mcl-1 by 1 mM SNP were reversed by 0.1 mM SNP pretreatment at the protein level by western blotting. Our study shows that priming with 0.1 mM SNP confers complete protection against cell death induced by 1 mM SNP in human articular chondrocytes. This protective effect was found to be correlated with the upregulation of both HO-1 and NF-κB and with the concomitant downregulation of both extracellular signal-regulated protein kinase 1/2 and p38 activation.

## Introduction

Articular cartilage consists of chondrocytes, the only cell type present, which are responsible for repairing tissue damage. Chondrocyte death and the pertinent signaling pathway involved have therefore been the focus of interest recently as pathogenetic factors leading to joint cartilage degradation in various forms of arthritides [1,2]. Several stimuli involved in the pathophysiology of arthritis, including nitric oxide (NO), Fas receptor ligation, and ceramide, have been reported to induce chondrocyte death *in vitro *[3-5].

The pathogenetic involvement of NO in arthritis was first demonstrated when levels of nitrite, a stable end product of NO metabolism, were shown to be elevated in serum and synovial fluid samples of rheumatoid arthritis patients and osteoarthritis patients [6]. Moreover, because osteoarthritic cartilage produces large amounts of NO, it could serve as a powerful initiator of chondrocyte death. In addition to the negative effects of NO on cartilage matrix synthesis (i.e. the inhibition of cartilage matrix macromolecule neosynthesis), the enhancement of matrix metalloproteinase activity, and the reduction of IL-1 receptor antagonist synthesis, NO may be an important mediator of cartilage degradation. However, the precise role of NO in the induction of chondrocyte death is debatable. For example, treatment with NO donors consistently induces cell death in cultured chondrocytes [3,7], whereas the production of high levels of endogenous NO by the overexpression of inducible NO synthase in transfected chondrocytes was not found to cause cell death [8]. This discrepancy may be attributed to the use of chemical NO donors, which not only generate reactive nitrogen species but also produce various secondary reactions depending on the cellular milieu *in vitro*. A recent study that employed diazeniumdiolates, which have been shown to be reliable sources of NO, demonstrated that exogenous NO is not cytotoxic to cultured chondrocytes *per se*, and that NO can even be protective under certain conditions of oxidative stress [9]. In addition, nitrite was found to exert a protective effect upon hypochlorous acid-induced chondrocyte toxicity, thus suggesting that NO has a novel cytoprotective role in inflamed joints [10].

This paradoxical effect of NO on cytotoxicity indicates that previous results using sodium nitroprusside (SNP) or *S*-nitroso-*N*-acetyl-L-penicillamine (SNAP) as NO donors should be cautiously interpreted. It has recently been reported that a low concentration of SNP exerts a protective effect against the cytotoxicity induced by higher concentrations of SNP, or against glucose deprivation in hepatocytes [11,12]. The objective of this study was to investigate the influence of low SNP concentrations upon the cytotoxicity induced by higher concentrations of SNP in chondrocytes. We also explored the mechanism of this low-concentration SNP-mediated cytoprotection.

## Materials and methods

### Reagents

Nitrate/nitrite colorimetric assay kits were purchased from Cayman Chemical (Ann Arbor, MI, USA). Ly83583 was purchased from Calbiochem (San Diego, CA, USA), 1-hydroxy-2-oxo-3-(3-aminopropyl)-3-isopropyl-1-triazene (NOC-5), SNAP, SB202190, PD98059, MG132 and Bay 11-7082 were from Alexis (Carlsbad, CA, USA), and zinc protoporphyrin (ZnPP) and cobalt protoporphyrin (CoPP) were from Frontier Scientific (Logan, UT, USA). Anti-phospho extracellular signal-regulated protein kinase (ERK) 1/2, anti-phospho-p38, anti-ERK 1/2, and anti-p38 were purchased from New England Biolabs (Beverly, MA, USA), anti-Bcl-2 from Transduction Laboratories (Lexington KY, USA), anti-Bax from Pharmingen (San Diego, CA, USA), and anti-heme oxygenase 1 (anti-HO-1), anti-Bcl-_XL_, anti-Mcl-1, anti-p53, anti-CIAP1 and anti-CIAP2 from Santa Cruz (Santa Cruz, CA, USA). All other reagents were obtained from Sigma (St Louis, MO, USA) unless specified otherwise.

### Chondrocyte culture

Cartilage samples were obtained from the femoral condyle and from the tibial plateau of the knees of osteoarthritis patients at the time of joint replacement surgery. All cartilage samples were procured after obtaining oral informed consent from the patients and institutional approval. Pieces of articular cartilage were cut, minced, and incubated sequentially with pronase and collagenase in DMEM until they had been digested. Released cells were seeded at 4 × 10^6^/plate in 10 cm culture plates in DMEM supplemented with 10% FCS, 1% L-glutamine, and 1% Fungizone (Gibco, Grand Island, NY, USA) and in DMEM supplemented with penicillin/streptomycin (150 units/ml and 50 mg/ml, respectively). Confluent chondrocytes were split once after about 7 days and were seeded at high density, and these first-passage adherent chondrocytes were then used in subsequent experiments.

### Nitrate/nitrite quantification

NOC-5 was dissolved in 10 mM NaOH to produce a 200 mM stock solution and was stored at -20°C. SNP, 3-morpholinosydnonimine (SIN-1), and SNAP were freshly dissolved in water before each experiment. All NO donor compounds were diluted with DMEM and added directly to cultured chondrocytes. The final products of NO *in vivo *are nitrite and nitrate, the sum of which can be used as an index of total NO production. Chondrocyte culture media were harvested after being incubated for 24 hours with the respective NO donors, and were then analyzed using a nitrate/nitrite colorimetric assay kit as recommended by the manufacturer. Briefly, nitrate was converted to nitrite using nitrate reductase, and then Griess reagents were added to form a deep-purple azo compound. Absorbance was measured at 540 nm using a plate reader to determine the nitrite concentrations. The detection limit of the assay was 1 μM.

### Quantification and verification of cell death

Cell death was quantitated using the 3-(4,5-dimethylthiazol-2-yl)-2,5 diphenyltetrazdium bromide (MTT) assay, as previously described [13]. Briefly, chondrocytes were seeded at 4 × 10^4^/100 μl/well in 96-well microtiter plates. Cell death was induced by treating with 1 mM SNP. To protect cells, chondrocytes were treated with 0.1 mM SNP, 50 μM CoPP, or 1 mM dibutylyl guanosine-3',5'-cyclic monophosphate (DBcGMP) 14 hours prior to being treated with 1 mM SNP. MTT was then added to each well to a final concentration of 0.125 mg/ml after they had been incubated with 1 mM SNP for 24 hours, and plates were incubated at 37°C for a further 3 hours. The formazan product obtained was solubilized with 100 μl dimethylsulfoxide and optical densities were read at 595 nm. Percentage cell survival was calculated by taking the optical density of cells post-treatment, dividing this by the optical density of the untreated control cells, and multiplying by 100. Cell death was also verified by flow cytometry. Chondrocytes were trypsinized after treatment and were sedimented, and the cell pellets obtained were washed and stained with 100 μg/ml propidium iodide solution for 15 min. For each sample, 10^4 ^cells were analyzed by FACS II flow cytometry (Becton Dickinson, Mountain View, CA, USA).

### Western blot

Cellular proteins were extracted in lysis buffer containing 50 mM sodium acetate, pH 5.8, 10% v/v SDS, 1 mM ethylene diaminetetraacetic acid, 1 mM phenylmethylsulfonyl fluoride, and 1 μg/ml aprotinin at 4°C. Samples were electrophoresed on 12% SDS-polyacrylamide gel, and transferred to polyvinylidene difluoride membranes. Blots were blocked with Tris-buffered saline containing 5% non-fat milk at room temperature for 1 hour, and then incubated with the respective antibodies overnight at 4°C. Finally, blots were incubated with 1:5000 peroxidase-conjugated goat anti-mouse or anti-rabbit IgG (Biorad, Hercules, CA, USA) for 1 hour. Bound immunoglobulin was detected by enhanced chemiluminescence (Amersham, Bucks, UK).

### Electrophoretic mobility shift assay

Nuclear extracts from chondrocytes were prepared from 2 × 10^6 ^cells, as described previously with minor modification [14]. Briefly, cells were incubated on ice for 15 min with homogenization buffer containing 10 mM HEPES-KOH, 4 mM MgCl_2_, 10 mM KCl, 1 mM NaF, 0.5 mM dithiothreitol, 1 mM phenylmethylsulfonyl fluoride, and 20 μg/ml leupeptin. After adding detergent, the lysates were centrifuged at 3000*g *for 5 min. Pellets were resuspended in extraction buffer containing 20 mM HEPES-KOH, 1.5 mM MgCl_2_, 420 mM NaCl, 25% glycerol, 1 mM NaF, 0.5 mM dithiothreitol, 1 mM phenylmethylsulfonyl fluoride, 20 μg/ml leupeptin, and 0.2 mM ethylene diaminetetraacetic acid. After incubation on ice and centrifugation, supernatants were collected, the protein content was measured, and 5 μg portions of extracts were used for the binding reaction. A consensus double-stranded NF-κB probe was obtained from Promega (Madison, WI, USA), and was end-labeled using γ-^32^P-adenosine-5-triphosphate. After incubating nuclear extracts in 2 μl gel binding buffer (Promega), end-labeled probe was added (100,000 cpm/sample). Samples were then incubated for 20 min and were loaded onto 4% nondenaturing polyacrylamide gels. Electrophoresis was run for 3 hours at 4°C. Protein complexes were identified by autoradiography.

### Data analysis

Data are expressed as means ± standard deviations. The paired *t *test was used to compare controls and treatment conditions, and significance was accepted at a confidence level of 95% (*P *< 0.05).

## Results

### Chondrocyte death does not correlate with the amount of NO released by NO donors

**Figure 1:**
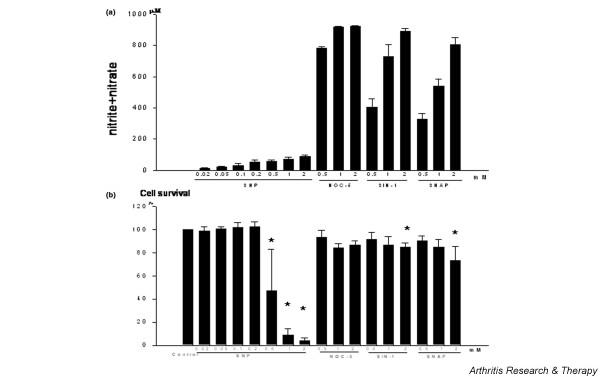
Quantification of nitrate/nitrite levels and the cell death induced by different nitric oxide (NO) donor compounds. Chondrocytes were obtained from the femoral condyle and the tibial plateau of knee osteoarthritis patients, cultured in monolayers, and seeded at 4 × 10^4^/100 μl/well in 96-well microtiter plates. First-passage chondrocytes were used in the subsequent experiments. Chondrocytes were treated with the respective NO donor compounds for 24 hours. **(a) **Chondrocyte culture media were harvested and analyzed with a nitrate/nitrite colorimetric assay kit, as described in Materials and methods. **(b) **Cell death was quantitated using the 3-(4,5-dimethylthiazol-2-yl)-2,5 diphenyltetrazdium bromide (MTT) assay. MTT was added to each well after the collecting medium. Percentage cell survival was calculated by dividing the optical density of treated cells by the optical density of untreated control cells, and multiplying by 100. Cell survival in control culture was set at 100%. * *P *< 0.05 versus control. Data shown are the means and standard deviations of duplicate experiments on three different donors. NOC-5, 1-hydroxy-2-oxo-3-(3-aminopropyl)-3-isopropyl-1-triazene; SIN-1, 3-morpholinosydnonimine; SNAP, *S*-nitroso-*N*-acetyl-L-penicillamine.

A nitrate/nitrite assay kit was used to determine the amount of NO generated by the various NO donor compounds, SNP, NOC-5, SIN-1, and SNAP [15]. As was reported previously [9], the different NO donors released variable degrees of NO in the culture medium; SNP was the least efficient NO producer (Fig. 1a). A one millimolar concentration of SNP yielded about 12% of the NO produced by 1 mM diazeniumdiolate, NOC-5. However, 1 mM SNP led to almost complete chondrocyte death, whereas the same concentration of NOC-5 caused no appreciable cell death 24 hours after treatment (Fig. 1b). Other NO donors, SIN-1 and SNAP, also induced significant chondrocyte death at 2 mM concentrations. The amounts of NO produced by 2 mM SIN-1 or 2 mM SNAP were 10-fold and 8.9-fold higher than that produced by 2 mM SNP, respectively, but the levels of cell death induced were not as profound as that produced by 2 mM SNP. These results demonstrate that the amount of NO produced by a NO donor is not correlated with chondrocyte death.

### 0.1 mM SNP protects chondrocytes from death induced by 1 mM SNP

**Figure 2:**
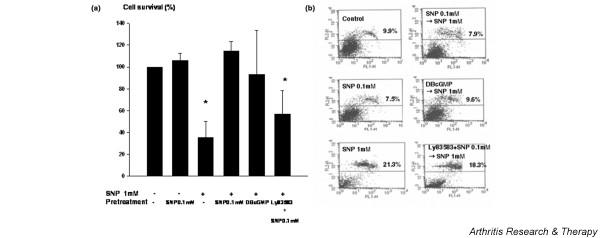
Protective effect of low-concentration sodium nitroprusside (SNP) on human articular chondrocytes. Cell death was induced by treating chondrocytes with 1 mM SNP for 24 hours. To protect them from cell death, chondrocytes were treated with 0.1 mM SNP or 1 mM dibutylyl guanosine-3',5'-cyclic monophosphate (DBcGMP) 14 hours prior to 1 mM SNP treatment. To inhibit cyclic guanylase, 1 μM Ly83583 was added with 0.1 mM of SNP. **(a) **Cell death was quantitated using the 3-(4,5-dimethylthiazol-2-yl)-2,5 diphenyltetrazdium bromide assay. Cell survival in control cultures was set at 100%. Data shown are the means and standard deviations of triplicate experiments from at least three different donors. * *P *< 0.05 versus control. **(b) **Cell death was verified by propidium iodide staining and fluorescence-activated cell sorting analysis. Chondrocytes were trypsinized after treatment and were sedimented. Cell pellets obtained were washed and stained in 100 μg/ml propidium iodide solution for 15 min. For each sample, 10^4 ^cells were analyzed. Data are representative of samples from four different donors. Percentage values denote propidium iodide positive (dead) chondrocytes.

It was previously reported that pretreatment of hepatocytes with a low dose of SNP significantly inhibited high-dose SNP-induced hepatocyte death [11,12]. In order to determine whether this phenomenon also occurs in chondrocytes, we treated chondrocytes with a low, noncytotoxic concentration of SNP (i.e. 0.1 mM). As shown in Fig. 2a, priming the chondrocytes with 0.1 mM SNP for 14 hours completely inhibited the cell death induced by 1 mM SNP. However, pretreatment with concentrations higher than 0.2 mM SNP did not confer protection (data not shown). Pretreatment with 0.1 mM SNP for 1–6 hours was also protective (data not shown), but because the degree of protection was greatest for the 14-hour pretreatment, chondrocytes were pretreated with 0.1 mM SNP for 14 hours in all subsequent experiments.

Inhibition of cell death was also verified by fluorescence-activated cell sorting analysis of treated chondrocytes stained with propidium iodide (Fig. 2b). Because low concentrations of SNP are known to protect a murine macrophage cell line via the cGMP signaling pathway [16], we investigated whether cGMP is also protective in chondrocytes. Chondrocytes were thus pretreated with 1 mM DBcGMP, a cell-permeable cGMP analog, for 14 hours before administering 1 mM SNP. As is shown in Fig. 2a, pretreatment with DBcGMP led to the complete inhibition of 1 mM SNP-mediated chondrocyte death. In addition, pretreatment with LY83583, a soluble guanylate cyclase inhibitor, negated the protective effect of 0.1 mM SNP pretreatment, thus implicating the cGMP pathway in 0.1 mM SNP-mediated chondrocyte cytoprotection.

### NOC-5 protects chondrocytes from 1 mM SNP-induced death

**Figure 3:**
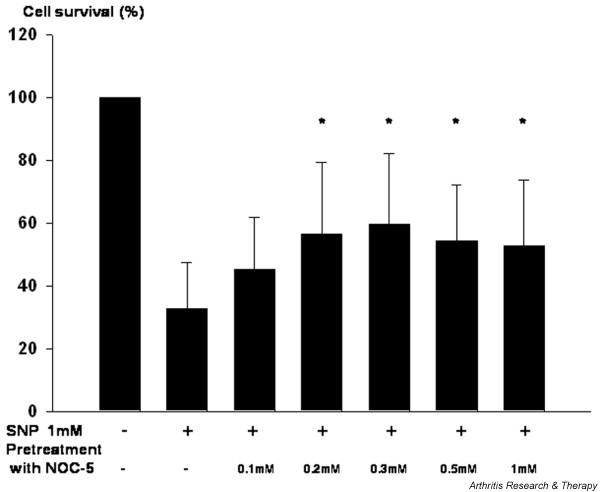
Effect of 1-hydroxy-2-oxo-3-(3-aminopropyl)-3-isopropyl-1-triazene (NOC-5) on the cytotoxic effect of 1 mM sodium nitroprusside (SNP) in human articular chondrocytes. Cell death was induced by treating chondrocytes with 1 mM SNP for 24 hours. To protect them from cell death, chondrocytes were treated with various concentrations of NOC-5 for 14 hours prior to 1 mM SNP treatment. Cell death was quantitated by the 3-(4,5-dimethylthiazol-2-yl)-2,5 diphenyltetrazdium bromide assay. Cell survival in control cultures was set at 100%. Data are the means and standard deviations of triplicate experiments on nine different donors. * *P *< 0.05 versus treatment with 1 mM SNP without NOC-5 pretreatment.

We also investigated whether 0.1 mM SNP-mediated cytoprotection is replicated by other NO donors. Low concentrations (0.1–0.5 mM) of SIN-1 or SNAP did not protect from the cell death induced by 1 mM SNP, but rather acted synergistically with SNP to enhance the cytotoxicity of subsequent 1 mM SNP treatment (data not shown). On the other hand, NOC-5 slightly inhibited 1 mM SNP-induced chondrocyte death, with maximal effect at 0.3 mM (Fig. 3).

### The protection conferred by low concentration SNP is mediated by HO-1 upregulation

**Figure 4:**
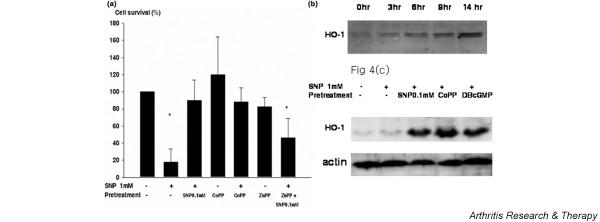
Protective effect of heme oxygenase 1 (HO-1) on human chondrocytes. **(a) **Cell death was induced by treating chondrocytes with 1 mM sodium nitroprusside (SNP) for 24 hours. For HO-1 induction, chondrocytes were treated with 50 μM cobalt protoporphyrin (CoPP) 14 hours prior to treating them with 1 mM SNP. For HO-1 inhibition, chondrocytes were treated with 1 μM zinc protoporphyrin (ZnPP) along with 0.1 mM SNP. Cell death was quantitated using the 3-(4,5-dimethylthiazol-2-yl)-2,5 diphenyltetrazdium bromide assay. Cell survival in control culture was set at 100%. Data shown are the means and standard deviations of triplicate experiments from at least four different donors. * *P *< 0.05 versus control. **(b) **Induction of HO-1 by 0.1 mM SNP treatment in human chondrocytes was analyzed by western blotting. Protein was extracted from chondrocytes after the indicated incubation periods and 20 μg each protein sample was separated by 12% SDS-PAGE and blotted with anti-HO-1 antibody. Data are representative of two samples from different donors. **(c) **Upregulation of HO-1 by pretreating chondrocytes with 0.1 mM SNP, 50 μM CoPP, or 1 mM dibutylyl guanosine-3',5'-cyclic monophosphate (DBcGMP). Chondrocytes were treated or not treated with the indicated chemicals for 14 hours and were then treated with 1 mM SNP for 2 hours. Protein was extracted from chondrocytes and 20 μg each protein sample was separated by 12% SDS-PAGE and blotted with anti-HO-1 antibody. The data shown are representative of five samples from different donors.

Because 0.1 mM SNP inhibited chondrocyte cytotoxicity induced by 1 mM SNP more so than the other NO donors examined, we investigated the mechanism of 0.1 mM SNP-mediated protection. Heme oxygenase is a rate-limiting enzyme in heme catabolism, and leads to the generation of bilirubin, free iron, and carbon monoxide (CO) [17-19]. HO-1 is inhibited by various metalloprotoporphyrins (e.g. ZnPP and tin protoporphyrin). Previous reports reveal that HO-1 overexpression is cytoprotective in multiple models including endotoxemia, shock, and ischemia/reperfusion [20-22]. Pretreatment of chondrocytes with the HO-1 inducer CoPP at 50 μM reproduced the cytoprotective effect of 0.1 mM SNP upon 1 mM SNP-induced cell death (Fig. 4a). Conversely, the co-treatment of chondrocytes with the HO-1 inhibitor ZnPP and 0.1 mM SNP attenuated the cytoprotective effect of 0.1 mM SNP. HO-1 was time-dependently induced by 0.1 mM SNP in chondrocytes, and western blot revealed that HO-1 is significantly upregulated after pretreatment with 0.1 mM SNP for 14 hours (Fig. 4b,c). CoPP at 50 μM upregulated HO-1 as was expected, and DBcGMP was also found to upregulate HO-1 in chondrocytes (Fig. 4c).

### The protection conferred by 0.1 mM SNP is correlated with the downregulation of ERK 1/2 and p38 kinase activation

**Figure 5:**
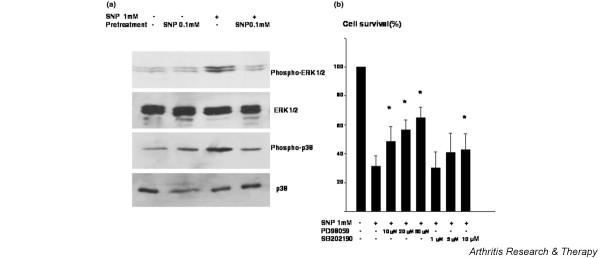
The regulation of extracellular signal-regulated protein kinase (ERK) 1/2 and p38 phosphorylation on 0.1 mM sodium nitroprusside (SNP) pretreated chondrocytes. **(a) **Chondrocytes were treated with 0.1 mM SNP 14 hours prior to treatment with 1 mM SNP and the phosphorylations of ERK 1/2 and p38 were analyzed after 4 hours by western blotting. Protein was extracted from chondrocytes and 20 μg each protein sample was separated by 12% SDS-PAGE and blotted with anti-phospho-ERK 1/2 or anti-phospho-p38. The expressions of ERK 1/2 and p38 were also determined by western blot analysis. Data are representative of samples from four different donors. **(b) **Effect of ERK 1/2 and p38 kinase inhibition on the chondrocyte death induced by 1 mM SNP. Cell death was induced by treating chondrocytes with 1 mM SNP for 24 hours. To inhibit ERK 1/2 or p38 kinase, chondrocytes were pretreated with 10, 20, or 50 μM PD98059 or with 1, 5, or 10 μM SB202190, respectively, for 2 hours before treating them with 1 mM SNP. Cell death was quantitated by the 3-(4,5-dimethylthiazol-2-yl)-2,5 diphenyltetrazdium bromide assay. Cell survival in control culture was set at 100%. Data are the means and standard deviations of duplicate experiments from at least three different donors. * *P *< 0.05 versus treatment with 1 mM SNP without pretreatment.

SNP at 1 mM caused the upregulation of both ERK 1/2 and p38 phosphorylation followed by chondrocyte death (Fig. 5a), and priming with 0.1 mM SNP reversed this pattern of mitogen-activated protein (MAP) kinase activation, by downregulating both ERK 1/2 and p38 phosphorylation. Pretreatment with the ERK 1/2 inhibitor PD98059 partially protected chondrocytes from death mediated by 1 mM SNP. The P38 kinase inhibitor SB202190 protected 1 mM SNP-mediated chondrocyte death only at 10 μM, which may inhibit pathways other than p38 (Fig. 5b). This result shows that the protection conferred by 0.1 mM SNP correlates with the downregulation of both ERK 1/2 and p38 kinase activation, but only the activation of ERK 1/2 was found to be directly responsible for chondrocyte death induced by 1 mM SNP.

### The protection conferred by 0.1 mM SNP is negated by NF-κB suppression

**Figure 6:**
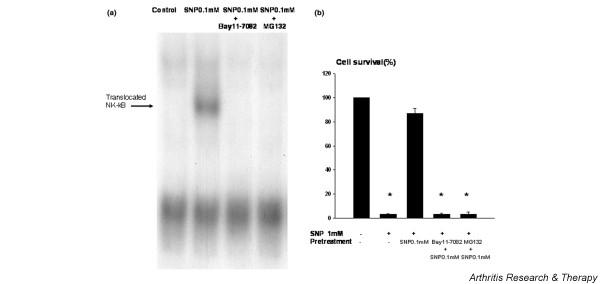
NF-κB activation was correlated with chondrocyte survival mediated by low-concentration sodium nitroprusside (SNP). **(a) **Activation of NF-κB in 0.1 mM SNP-treated chondrocytes. Chondrocytes were treated with 0.1 mM SNP for 14 hours with or without NF-κB inhibitors and the activation of NF-κB was analyzed by electrophoretic mobility shift assay. Nuclear extracts were prepared from 2 × 10^6 ^cells, and 5 μg portions of extracts were used for the binding reaction. Nuclear extracts were incubated in gel binding buffer with radiolabeled consensus double-stranded NF-κB probe, and samples were loaded onto 4% nondenaturing polyacrylamide gel. Protein complexes were identified by autoradiography. Data are representative of three samples from different donors. **(b) **Effect of the inhibition of NF-κB activation on the protective effect of 0.1 mM SNP on human chondrocytes. Cell death was induced by treating chondrocytes with 1 mM SNP for 24 hours. To inhibit NF-κB, chondrocytes were co-treated with 20 μM Bay 11-7082 or MG132 and 0.1 mM SNP for 14 hours before treating with 1 mM SNP. Cell death was quantitated by the 3-(4,5-dimethylthiazol-2-yl)-2,5 diphenyltetrazdium bromide assay. Cell survival in the control culture was set at 100%. Data are the means and standard deviations of triplicate experiments from at least three different donors. * *P *< 0.05 versus control.

Because NF-κB activation plays a pivotal role in protecting chondrocytes from apoptosis induced by death signals [23,24], the role of NF-κB activation in the protective effect of 0.1 mM SNP was examined. Activation of NF-κB by 0.1 mM SNP pretreatment was verified by electrophoretic mobility shift assay (Fig. 6a). Co-treatment with the NF-κB inhibitor Bay 11-7082 and with 0.1 mM SNP completely negated the protective effect of 0.1 mM SNP (Fig. 6a). Because Bay 11-7082 was found to be cytotoxic to chondrocytes (data not shown), another NF-κB inhibitor MG132, which is not cytotoxic to chondrocytes, was also tested. It was found that MG132 co-treatment also negated the protection conferred by 0.1 mM SNP. This result implies that NF-κB activation participates in the chondrocyte protection mediated by 0.1 mM SNP.

### The protection conferred by 0.1 mM SNP correlates with the upregulation of Bcl-2 family proteins and the downregulation of p53

**Figure 7:**
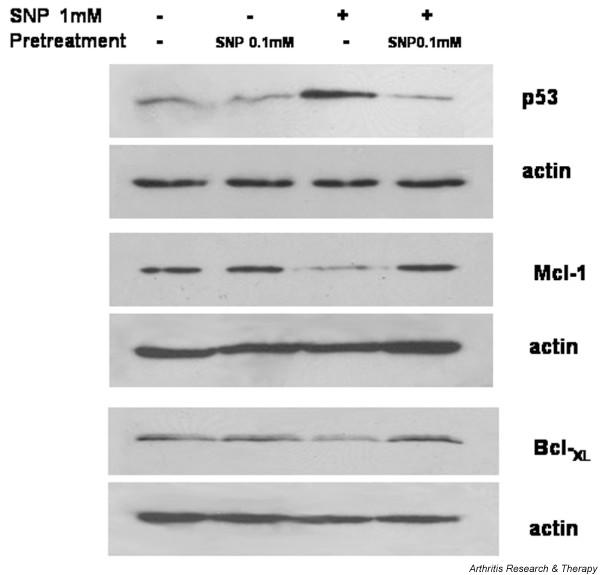
Regulation of apoptosis-related proteins in sodium nitroprusside (SNP)-treated chondrocytes. Chondrocytes were treated with 0.1 mM SNP 14 hours prior to being treated with 1 mM SNP, and the expressions of Mcl-1, Bcl-XL, and p53 were analyzed after 4 hours by western blot. Protein was extracted from chondrocytes and 20 μg each protein sample was separated by 12% SDS-PAGE and blotted with respective antibodies. Data are representative of five samples from different donors.

The Bcl-2 family proteins MCl-1 and Bcl-_XL _were both downregulated during the cell death induced by 1 mM SNP (Fig. 7). This downregulation was reversed by priming chondrocytes with 0.1 mM SNP. On the contrary, p53 was upregulated during 1 mM SNP-mediated chondrocyte death, but was downregulated by 0.1 mM SNP pretreatment (Fig. 7). The expressions of other Bcl-2 family members, such as Bcl-2 and Bax, or of the IAP family, c-IAP1, c-IAP2, or XIAP, were unaffected (data not shown).

## Discussion

The mechanism of SNP-mediated chondrocyte death has been extensively investigated, and has usually been viewed as a NO-mediated form of chondrocyte apoptosis. In line with a previous result [9], our result shows that SNP is the least potent in terms of producing exogenous NO in chondrocyte culture, yet it is the most potent inducer of chondrocyte death. We cannot rule out the role played by NO in SNP-mediated chondrocyte death, because it is not possible to quench the NO produced by SNP treatment selectively. However, it is believed unlikely that NO is the sole mediator of SNP-induced chondrocyte death and peroxynitrite, a reaction product of NO and superoxide anions, or the primary byproducts of the decomposition of SNP, such as the cyanide anion or pentacyanoferrate complex, might contribute to its cytotoxicity [25,26].

Cytotoxic concentrations of SNP are associated with a 20-fold increase in NO production versus noncytotoxic concentrations, which contrasts with the actions of other nontoxic NO donors, which increase NO concentrations several hundred fold. It was of interest to find that pretreatment with 0.1 mM SNP led to complete chondrocyte protection against the toxic effect of 1 mM SNP. NOC-5, a diazeniumdiolate, also inhibited 1 mM SNP-induced chondrocyte death. However, despite the much higher level of NO formed by NOC-5, the degree of protection it conferred was smaller than that conferred by 0.1 mM SNP. It is thus also likely that the protection conferred by low-concentration SNP is not solely explained by NO production. It remains for further research to identify the other cytoprotective component mediated by low-concentration SNP.

In the present study, we used chondrocytes obtained from osteoarthritis patents at the advanced stage, because it was not possible to obtain sufficient chondrocytes from normal cartilage to carry out the *in vitro *experimentation. Although it is not possible to extrapolate our results to normal chondrocytes, a limited experiment utilizing normal cartilage obtained from a femoral head revealed that 0.1 mM SNP protected chondrocytes from cell death induced by 1 mM SNP to the same degree as was observed in osteoarthritis chondrocytes (data not shown).

To elucidate the signaling mechanism involved in low-concentration SNP-mediated cytoprotection, cGMP dependence was first examined. In the present study, the soluble guanylate cyclase inhibitor LY83583 was found to inhibit the cytoprotective effect of 0.1 mM SNP, whereas DBcGMP, a cell-permeable cGMP analog, attenuated the cell death induced by 1 mM SNP, indicating a cGMP-mediated cytoprotective mechanism. A previous study showed that the protection afforded by DBcGMP against SNP-induced death in RAW264 cells is mediated by protein kinase G activation, which results in the inhibition of cytochrome c release [16]. Because the inhibition of cytoprotection by Ly83583 was incomplete in the present study, and because other NO donors such as SIN-1 and SNAP, which also induce cGMP, failed to protect chondrocytes, other cytoprotective pathways were also examined.

Recent evidence has demonstrated the critical importance of HO-1 expression in the mediation of antioxidant, anti-inflammatory, and anti-apoptotic effects [19,27,28]. HO-1 is distributed ubiquitously and is induced strongly by a variety of physiologic and pathophysiologic stimuli, including heme, heavy metals, inflammatory cytokines, endotoxins, and NO [12]. The pretreatment of chondrocytes with the HO-1 inducer CoPP reproduced the cytoprotective effect of 0.1 mM SNP against 1 mM SNP-induced cell death, whereas the co-treatment of chondrocytes with the HO-1 inhibitor ZnPP and 0.1 mM SNP inhibited this cytoprotective effect. Moreover, HO-1 was found to be induced by 0.1 mM SNP treatment in chondrocytes.

The mechanism by which HO-1 protects from cell death has been postulated to involve several mechanisms, although the role of CO produced by the HO-1 degradation of heme has received most attention. Pharmacologic CO donors have also been demonstrated to protect hepatocytes from the death induced by glucose deprivation or anti-Fas [29]. Zuckerbraun and colleagues [30] recently showed that CO mediates hepatocyte protection by activating NF-κB, which in the presence of an inflammatory stimulus upregulates inducible NO synthase and leads to NO production. This mechanism implies a synergy between CO and NO in the provision of cytoprotection. Increased HO-1 activity also results in the generation of bilirubin, an antioxidant capable of scavenging peroxy radicals and inhibiting lipid peroxidation [29]. Finally, ferritin is another catalytic byproduct of HO-1 induction, and sequesters the free iron produced during heme catalysis, which reduces intracellular free iron and thus has an anti-oxidant effect [31]. The downstream process of cytoprotection conferred by the upregulation of HO-1 in human chondrocytes warrants further study. HO-1 was recently detected in human cartilage and in chondrocytes, and was found to be downregulated by proinflammatory cytokines and to be upregulated by anti-inflammatory cytokine, suggesting that HO-1 is a component of the protective mechanisms in human cartilage [32].

In a previous report, cell death and the dedifferentiation of chondrocytes was demonstrated to be regulated oppositely by two MAP kinase subtypes, ERK 1/2 and p38 kinase [33]. In rabbit chondrocytes, SNP increased both p38 kinase and ERK activation, and SNP-induced p38 kinase functioned as an induction signal for apoptosis and in the maintenance of the chondrocyte phenotype, whereas ERK activity caused dedifferentiation and operated as a prosurvival signal. Although our results show that high-dose SNP induces both p38 and ERK phosphorylation in line with the previous report [33], the downregulation of ERK 1/2 phosphorylation by low-concentration SNP was associated with chondrocyte protection rather than cell death in our human chondrocyte cultures.

The role played by ERK inhibition in chondrocyte death is not without controversy. Whereas one report showed that the blocking of MAP kinase kinase upstream of ERK by U0126 induces chondrocyte death, another report showed that ERK 1/2 or p38 kinase inhibition prevents SNP-induced chondrocyte death [34,35]. We found that the inhibition of ERK 1/2 leads to partial protection against 1 mM SNP-mediated chondrocyte death, but SB202190 at low concentrations, which specifically suppresses p38 activation, did not suppress it. This discrepancy probably stems from the differences in culture conditions, and concentrations of the inhibitors used. According to our result, although the protection conferred by 0.1 mM SNP correlates with the downregulation of both ERK 1/2 and p38 kinase activation, only the activation of ERK 1/2 is directly responsible for chondrocyte death induced by 1 mM SNP.

Finally, the role played by NF-κB activation in 0.1 mM SNP-mediated chondrocyte protection was investigated because NF-κB has been reported to serve as a survival signal in both tumor necrosis factor alpha and anti-Fas-mediated chondrocyte death [23,24]. NF-κB activation was observed after pretreating 0.1 mM SNP in human chondrocytes. Pretreating with the NF-κB inhibitors MG132 or Bay 11-7085 completely abolished the protection conferred by 0.1 mM SNP. Because this inhibition of the protective effect of SNP was greater than that conferred by either HO-1 or cyclic guanylase inhibitor, we believe that NF-κB has a pivotal role in the protective mechanism signaled by low-dose SNP in chondrocytes.

Of the regulators of cell survival, the expressions of p53, Bcl-_XL_, and Mcl-1 were significantly affected by 0.1 mM SNP pretreatment. The upregulation of p53 induced by 1 mM SNP was downregulated by 0.1 mM SNP pretreatment. Although we did not determine the mechanistic role of p53 phosphorylation, it is generally recognized that the phosphorylation of p53 leads to its accumulation, and that p53 is phosphorylated either indirectly or directly by c-Jun N terminal kinase, by p38 kinase, or by ERK [36-38]. We hypothesize that 1 mM SNP induced p38 kinase and ERK activity in chondrocytes and phosphorylated p53, resulting in p53 accumulation, and that this was negated by 0.1 mM SNP pretreatment via the downmodulation of these MAP kinases. Of the Bcl-2 family members, the downregulations of Bcl-_XL _and Mcl-1, both anti-apoptotic species, by 1 mM SNP was reversed by 0.1 mM SNP.

Despite the marked improvements made in our understanding of the mechanisms of chondrocyte apoptosis over the past several years, it is unclear whether chondrocyte apoptosis is the major mechanism of cartilage degradation or merely a byproduct of tissue degeneration. Thus, whether the modulation of apoptosis represents a feasible therapeutic target for the treatment of osteoarthritis is not obvious at the moment. A recent report showing that the intra-articular instillation of the pan-caspase inhibitor zVAD-fmk into the knees of rabbits induced to osteochondral injury led to a significant reduction in chondrocyte apoptosis implies that apoptosis inhibitors could be used to inhibit chondrocyte death in traumatic cartilage injury [39].

## Conclusion

The present study shows that the widely used NO donor SNP at 1 mM concentration mediates chondrocyte death strongly despite its relatively poor ability to produce NO compared with other NO donors. Pretreating chondrocytes with SNP at 0.1 mM (a noncytotoxic concentration) protects the cells against 1 mM SNP cytotoxicity. This protective pathway was found to be related to four factors: cyclic GMP, HO-1, MAP kinase, and NF-κB. The study elucidates the survival pathway inherent in chondrocytes, and provides strategic information for the development of new therapeutics based on the regulation of chondrocyte death

## Abbreviations

CO = carbon monoxide; CoPP = cobalt protoporphyrin; DBcGMP = dibutylyl guanosine-3',5'-cyclic monophosphate; DMEM = Dulbecco's modified Eagle's medium; ERK = extracellular signal-regulated protein kinase; FCS = fetal calf serum; HO-1 = heme oxygenase 1; IL = interleukin; MAP = mitogen-activated protein; MTT = 3-(4,5-dimethylthiazol-2-yl)-2,5 diphenyltetrazdium bromide; NF = nuclear factor; NO = nitric oxide; NOC-5 = 1-hydroxy-2-oxo-3-(3-aminopropyl)-3-isopropyl-1-triazene; SIN-1 = 3-morpholinosydnonimine; SNAP = *S*-nitroso-*N*-acetyl-L-penicillamine; SNP = sodium nitroprusside; ZnPP = zinc protoporphyrin.

## Competing interests

The author(s) declare that there are no competing interests.

## Authors' contributions

HAK conceived of the study, participated in its design, and supervised the experimental procedure. KBL provided samples, participated in the design of the study, and drafted the manuscript. S-cB performed the data analysis and drafted the manuscript.
